# Urodynamics post stroke in patients with urinary incontinence: Is there correlation between bladder type and site of lesion?

**DOI:** 10.4103/0972-2327.53078

**Published:** 2009

**Authors:** Anupam Gupta, Arun B. Taly, Abhishek Srivastava, Murali Thyloth

**Affiliations:** Department of Psychiatric and Neurological Rehabilitation, National Institute of Mental Health and Neuro Sciences (NIMHANS), Bangalore, India; 1 Department of Neurology, National Institute of Mental Health and Neuro Sciences (NIMHANS), Bangalore, India; 2 Presently at Department of Rehabilitation, Kokilaben Dhirubhai Ambani Hospital, Mumbai, India

**Keywords:** Stroke, urinary incontinence, urodynamic study

## Abstract

**Objective:**

Assessment of bladder by urodynamic study (UDS) in patients with urinary incontinence following stroke, and correlation with site of lesion.

**Study Design and Setting:**

Retrospective cross-sectional study in the neurological rehabilitation unit of a tertiary care institute.

**Materials and Methods:**

Forty patients (22 males) with arterial or venous, ischemic or hemorrhagic stroke, with urinary incontinence in the acute phase following the event, underwent UDS. Seventeen patients had right hemiplegia, 18 had left hemiplegia, and five had posterior circulation stroke with brainstem/cerebellar features. Bladder type was correlated with age, side, and site of lesion.

**Results:**

The mean age was 46.80 ± 16.65 years (range: 18-80 years). Thirty-six patients had arterial stroke and four had cortical venous thrombosis. UDS was performed after a mean of 28.32 ± 10.27 days (range: 8-53 days) after the stroke. All but one patient had neurogenic bladder dysfunction, with 36 patients (90%) having overactive detrusor (OD) and three having underactive/areflexic detrusor. Among the 36 patients with OD, 25 patients (62.5%) had OD without detrusor-sphincter dyssynergy (DSD) and 11 (27.5%) had OD with DSD. Bladder management was advised based on the UDS findings. No significant correlation (*P* > 0.05) was found between type of bladder and age or side and site of lesion.

**Conclusions:**

UDS is a useful tool to assess and manage the bladder following stroke with urinary incontinence. In this study, no significant correlation was found between UDS findings and site of lesion.

## Introduction

The prevalence of urinary incontinence is 38-60% in stroke survivors in the acute phase.[1–3] It is correlated with the size of the infarct or hemorrhage, the site of the lesion, the presence of cognitive impairment, aphasia, the morbidity & mortality, and the discharge destination of the patient.[4,5] Urinary incontinence has prognostic significance post stroke; patients with urinary incontinence have high mortality, with 52% dying within 6 months of the stroke.[6] Its presence is also a predictor of moderate or severe disability at 3 months post stroke in patients younger than 75 years of age.[7]

Gelber *et al.*[4] suggested three major mechanisms responsible for post-stroke urinary incontinence: Disruption of neuromicturition pathways (overactive detrusor and urge incontinence); stroke-related cognitive and language deficits, with normal bladder function; and concurrent neuropathy or medication use (underactive detrusor and overflow incontinence).[4]

Flisser *et al*.[8] divided patients with urinary complaints following stroke into four clinical categories:

- Type 1 - no evidence of involuntary detrusor contractions on videourodynamics
- Type 2 - involuntary detrusor contractions present, patient is aware of these contractions and able to abort them
- Type 3 - involuntary detrusor contractions present, patient aware of the contractions and able to contract the sphincter but not abort the contractions
- Type 4 - involuntary detrusor contractions present, patient unaware of the contractions and unable to contract the sphincter or abort the contractions.

Overactive detrusor (OD) has been reported to be the most common urodynamic finding in stroke patients in the acute phase.[9] Earlier studies have reported an association of OD with frontal lobe, basal ganglia, and internal capsule lesions, with or without detrusor- sphincter dyssynergy (DSD).[10,11] Aybek *et al*. suggested that urinary incontinence after a cerebrovascular accident is a reversible condition and is due to an imbalance between cortical and pontomesencephalic centers.[12]

This study was performed to evaluate the bladder in stroke patients with urinary incontinence in the acute phase by urodynamic study (UDS) and correlate the UDS findings with the site of lesion in brain.

## Materials and Methods

This retrospective cross-sectional study was performed in 40 stroke patients (22 males, 18 females) in the neurological rehabilitation unit of a tertiary care institute. Medical records of patients admitted in the stroke unit during January 2002-June 2006 and referred for UDS were traced. Patients with first or recurrent arterial or venous stroke, with complaints of urinary incontinence post episode, were included in the study. Patients with premorbid history of obstructive or irritative urinary complaints, prostate-related complaints, obstructive urinary complaints (post episode), and patients with altered sensorium and global aphasia were excluded from the study.

Age, gender, type of stroke (arterial *vs* venous), side of stroke (right *vs* left), and risk factors for stroke were noted. Time lapse between the stroke episode and UDS investigation (in days) and the radiological findings (CT or MRI scan of brain) of all patients were noted.

UDS was performed in all patients using multi-channel pressure recording technology, using Phoenix MK (Allbyn Medical, Scotland, UK) equipment. It consisted of filling and voiding cystometry, pressure profilometry, and measurement of residual urine. Cystometry was performed with normal saline (0.9%) at a medium fill rate (10-100 ml/min). Detrusor pressure during the filling and voiding phase, intravesical and abdominal pressure, and cystometric capacity of the bladder and compliance were recorded. Leak point pressure and dyssynergy (when observed) were also recorded. Patients were advised behavioral, supportive, or/and pharmacological measures, based on the study findings.

Bladder type (according to UDS findings) was correlated with site of lesion in the brain (according to the radiological findings), age of the patient, and side of lesion.

### Data analysis

Statistical analysis was done using SPSS, version 15.0. Descriptive statistics including frequencies, means and standard deviation for quantitative variables like age, risk factors, duration of illness, bladder type (according to UDS findings), and site of lesion were estimated. Nonparametric test (Spearman correlation coefficient) was used to assess the correlation between type of bladder (according to UDS) and site of lesion (by CT or MRI findings), age, and side of lesion.

## Results

The ages of the study subjects ranged from 18-80 years (mean: 46.80 ± 16.65). Thirty-six patients (90%) had arterial stroke (27 ischemic and 9 hemorrhagic) and four (10%) had venous stroke. Left hemiplegia was observed in 18 patients, while 17 patients had right hemiplegia.

Twenty-two patients had hypertension alone and 15 patients had diabetes alone, while 10 patients had both hypertension and diabetes. Three patients each were known smokers and alcohol users. All those with venous strokes were females: Three patients developed paralysis in the postpartum period and one patient was taking oral contraceptive pills for the last 2 years. One arterial stroke patient was a known case of rheumatic heart disease.

The radiological findings of all patients are listed in Table 1.

**Table 1:** Bladder according to urodynamic study and site of lesion in brain

**Site of lesion**	**Type of bladder according to urodynamic study**			
	
	**Overactive detrusor without sphincterdyssynergy**	**Overactive detrusor with sphincterdyssynergy**	**Underactive detrusor**	**Normal study**
Lt. basal gang.	-	3	1	-
Lt. frontopariet.	3	-	-	-
Lt. internal cap.	4	1	-	-
Multi-infarct	-	1	-	-
Lt. fr-par-temp.	2	-	-	-
Lt. thalamic	2	-	-	-
Rt. basal gang.	3	3	-	-
Rt. fronto-par.	3	1	-	1
Rt. tempor-par.	-	-	1	-
Rt. thalamic	1	2	-	-
Rt. frontal	1	-	-	-
Rt. internal cap.	2	-	-	-
Vert.-basil. insufficiency	4	-	1	-
Total	25	11	3	1

Lt.= left; Rt.= right; Basal gang. = basal ganglia; Frontopariet = frontoparietal lobe; Internal capsule. = internal capsule; Fr-par-temp. = frontoparieto-temporal lobe; Tempor-par. = temporo-parietal lobe; Vert.-basil. insuf = vertebro-basilar insufficiency

UDS was performed after a mean of 28.32 ± 10.27 days (range: 8-53 days) after the event. Mean cystometric capacity of the bladder in this study was 262.07 ± 176.41 ml. (range: 18-540). Thirty-six patients (90%) had OD in the study, of whom 25 (62%) had OD without DSD, 11 had OD with DSD, three patients (7.5%) had underactive detrusor, and one patient had a normal study. Based on the UDS findings, 30 patients (75%) were advised antimuscarinic medications (probanthine - 14, oxybutynin - 6, or tolterodine - 10 patients) on a daily basis. In addition, 27 patients were advised clean intermittent catheterization/self-catheterization, with fluid restriction and timed voiding. Thirteen patients were advised voluntary micturition, with or without medication. The response to all three antimuscarinic medications was excellent, with all patients reporting reduced frequency and urgency of micturition. The urge incontinence also decreased/stopped in patients. This favorable outcome was a result of the pharmacological, supportive, and behavioral management of bladder.

Dry mouth was reported by most of the patients after starting medication, especially with probanthine. Most of the patients did not continue to have the problem after a week or so of continuous medication. They were advised supportive measures. None of the patients reported hallucinations with antimuscarinics in this study.

Type of bladder (according to UDS) was correlated with site of lesion (according to radiological findings). It was found to be insignificant using nonparametric test (*P* = 0.586). Correlation between type of bladder and age and side of lesion was also observed to be insignificant (*P* = 0.609 and 0.179, respectively) [Table 1].

## Discussion

The bladder is innervated by both parasympathetic and sympathetic nerves, both of which are also controlled by higher cortical and subcortical centers. The detrusor muscle is innervated by parasympathetic nerves, while the internal sphincter is innervated by sympathetic nerves. The external sphincter is composed of striated muscle fibers and is innervated via the pudendal nerves. The micturition center in the pontomesencephalic tegmentum of the periaqueductal region receives afferent impulses from the sacral cord segments. Efferent fibers from the center course downward in the reticulospinal tracts in the lateral funiculi of the spinal cord. The micturition center also receives fibers from the anteromedial parts of the frontal cortex, limbic regions, amygdaloid nuclei, thalamus, hypothalamus, and cerebellum.[13,14]

It is believed that both the cerebral cortex and the basal ganglia have inhibitory effect on micturition, and dysfunction of these structures would cause OD.[15] Considering the neurophysiology, all patients with suprapontine lesions should have OD without sphincter dyssynergy and patients with a suprasacral infrapontine lesion should have OD with sphincter dyssynergy; however, this pattern has not been observed in most of the studies including ours.

In the present study, 90% of the patients had OD in the acute phase post stroke, and 62.5% patients had no sphincter dyssynergy. OD with uninhibited bladder contraction has been reported as being the most common urodynamic finding (68-90%) following a stroke in earlier studies also.[9,11,16,17] No significant correlation was found in the present study between site of lesion and type of bladder according to UDS (*P* = 0.586), although 90% of the patients had OD. In their study with stroke patients, Khan *et al*. (1990)[10] also observed similar findings, with the majority of the patients with cerebral cortex and/or internal capsule lesions having uninhibited relaxation of the sphincter during involuntary bladder contractions, while all patients with lesions only in the basal ganglia or thalamus had normal sphincters. However, according to the authors, correlation of bladder dysfunction with the area of brain injury was not conclusive. Sakakibura *et al.* (1996)[11] in their study with stroke patients observed OD in lesions of the frontal lobe as well as of the basal ganglia, uninhibited sphincter relaxation with lesions of the frontal lobe, and DSD with lesions of the basal ganglia. The authors suggested that the anteromedial frontal lobe and its descending pathway, and the basal ganglia, are mainly responsible for supranuclear types of pelvic and pudendal nerve dysfunction.

No significant correlation was found in the present study between type of bladder and age or side of lesion. Gelber *et al.* (1993)[4] in their series with stroke patients also reported that there was no significant correlation between bladder type and age or side of lesion.

### Limitations of the study

There are limitations in this study, the foremost being the small sample size. This was a cross-sectional study; a follow-up study would have given better information about the long-term consequences of stroke on the bladder and about the reversibility of the bladder complaints, mortality, and disability due to persistent urinary incontinence. As most of the patients were not admitted in the rehabilitation unit, correlation of bladder complaints with neurological and functional recovery could not be made.

## Conclusions

Patients with urinary incontinence following stroke should be assessed with UDS for management of neurogenic bladder. In the present study, most of the patients had OD, with or without DSD. No significant correlation was found between type of bladder and site of lesion, age, or side of lesion in this study.
